# Combined Citrulline and Glutathione Supplementation Improves Endothelial Function and Blood Pressure Reactivity in Postmenopausal Women

**DOI:** 10.3390/nu15071557

**Published:** 2023-03-23

**Authors:** Arturo Figueroa, Arun Maharaj, Yejin Kang, Katherine N. Dillon, Mauricio A. Martinez, Masahiko Morita, Dai Nogimura, Stephen M. Fischer

**Affiliations:** 1 Department of Kinesiology and Sport Management, Texas Tech University, Lubbock, TX 79409, USA; 2 Department of Epidemiology and Cancer Control, St. Jude Children’s Research Hospital, Memphis, TN 38105, USA; 3 Research & Development Division, KIRIN Central Research Institute, Kirin Holdings Co., Ltd., 2-26-1, Muraoka-Higashi, Fujisawa 251-8555, Kanagawa, Japan

**Keywords:** arginine/ADMA ratio, blood pressure responsiveness, citrulline, endothelial function, glutathione

## Abstract

Postmenopausal women (PMW) may experience endothelial dysfunction associated with arginine (ARG) deficiency relative to asymmetric dimethylarginine (ADMA) caused by oxidative stress. Endothelial dysfunction contributes to increased blood pressure (BP) responsiveness to sympathoexcitation induced by the cold pressor test (CPT). We investigated the effects of citrulline alone (CIT) and combined with the antioxidant glutathione (CIT+GSH) on vascular function. Forty-four healthy PMW were randomized to CIT (6 g), CIT+GSH (2 g + 200 mg: Setria^®^) or placebo (PL) for 4 weeks. Brachial artery flow-mediated dilation (FMD), aortic stiffness (pulse wave velocity, PWV), brachial and aortic BP reactivity to CPT, and serum fasting blood glucose (FBG), ARG, and ARG/ADMA ratio were measured. Baseline FBG was higher in CIT+GSH vs. PL. FMD increased after CIT+GSH vs. PL (*p* < 0.05). CIT and CIT+GSH increased ARG/ADMA (*p* < 0.05), but did not affect aortic PWV. CIT+GSH attenuated the brachial and aortic systolic BP and mean arterial pressure (MAP) responses to CPT vs. PL and CIT (*p* < 0.05). The improvements in FMD were related to baseline FMD (*r* = −0.39, *p* < 0.05) and aortic MAP response to CPT (*r* = −0.33, *p* < 0.05). This study showed that CIT+GSH improved FMD and attenuated systolic BP and MAP reactivity in PMW. Although CIT increased ARG/ADMA, it did not improve FMD in healthy PMW.

## 1. Introduction

The endothelium has a major role in the regulation of vascular function and structure by releasing vasodilatory molecules [1]. Nitric oxide (NO), the main vasodilator, is produced from the amino acid L-arginine (ARG) by endothelial NO synthase (eNOS) [2] in response to increased shear stress induced by blood flow. NO is the primary endothelial factor for relaxing vascular smooth muscle cells and preventing pathological structure remodeling that leads to arterial stiffness [1,3]. Reduced NO availability, a main cause of endothelial dysfunction [1,3], is associated with ARG deficiency driven by increased levels of endogenous inhibitors of eNOS [4] and ARG catabolism to ornithine (ORN) by the enzyme arginase [5,6]. Oxidative stress is a main mechanism of endothelial dysfunction via increasing asymmetric dimethylarginine (ADMA, an eNOS inhibitor) levels [7,8,9] and arginase activity [10]. Since ARG and ADMA compete for binding to eNOS, a low ARG/ADMA ratio is a biomarker of reduced ARG availability and NO production [7].

Impaired endothelial function, assessed as low brachial artery flow-mediated dilation (FMD), begins before and progresses after menopause in healthy women [4,11]. Endothelial dysfunction precedes the development of arterial stiffness [12] and hypertension in healthy postmenopausal women [13]. Another mechanism for hypertension in postmenopausal women is elevated sympathetic activity [14]. Systolic hypertension and consequent heart failure with preserved ejection fraction are highly prevalent in older women due to increased aortic stiffness [15] and sympathetic-mediated vasoconstriction [16,17]. Evidence indicates that augmented systolic blood pressure (BP) reactivity to a sympathetic stimulus (cold pressor test, CPT) predicts future hypertension [18] and heart failure [19]. Exaggerated BP reactivity to sympathetic stimuli seen in postmenopausal women [16], may be due to the inability of the endothelium to counteract sympathetic-mediated vasoconstriction [17,20].

Dietary strategies to increase circulating ARG may improve vascular function. The effectiveness of short-term oral ARG supplementation for improving FMD was observed in healthy older adults [21]. However, oral ARG becomes ineffective for vascular benefits due to stimulation of arginase activity [22]. Contrary to ARG, citrulline (CIT) is not catabolized by arginase and inhibits arginase activity [23], and thus, is more efficient than oral ARG in increasing circulating ARG levels [24]. Short-term CIT supplementation has been shown to improve plasma ARG, ARG/ADMA, arterial stiffness, and resting BP in middle-aged and older adults [23,25,26,27,28,29]. Evidence supports the benefit of CIT supplementation on the attenuation of BP reactivity to CPT in healthy adults [30,31,32]. A placebo-controlled study examined the effect of CIT supplementation on FMD but failed to detect improvements in healthy adults [24]. Since oxidative stress is implicated in endothelial dysfunction, the addition of the antioxidant glutathione (GSH) may synergize the vascular benefits of CIT. Recent evidence supports the combined use of CIT and GSH (CIT+GSH) to improve eNOS activity in a mouse ischemia model [33] and the long-lasting increase of plasma NO levels in young men [34]. Although combined CIT+GSH supplementation increased post-exercise plasma NO metabolites (NOx), an indicator of improved endothelial function [34], vascular function measures were not evaluated. Therefore, it is unknown whether CIT+GSH supplementation can improve vascular function and BP reactivity in postmenopausal women. Given that CIT supplementation increased circulating ARG levels and the ARG/ADMA ratio, an indicator of increased NO availability [27], improvements in brachial FMD are expected in postmenopausal women.

The purpose of this study was to investigate the effects of supplementing with CIT alone and CIT+GSH on vascular function, assessed by brachial FMD, arterial stiffness (pulse wave velocity, PWV), and BP reactivity to CPT in postmenopausal women. Moreover, we assessed fasting blood glucose (FBG), serum levels of ARG and its metabolites, and markers of oxidative stress. Our hypothesis was that CIT alone and CIT+GSH supplementations would increase brachial FMD and attenuate BP responses to sympathetic activation induced by CPT compared to a placebo in postmenopausal women.

## 2. Materials and Methods

### 2.1. Participants

Participants were healthy postmenopausal women aged 51–74 years. All participants had absence of menstruation for at least 1 year and were sedentary (<120 min/week of exercise). Potential participants were excluded if they had diagnosed cardiovascular diseases (CVDs), type 2 diabetes, a body mass index > 34.9 kg/m^2^, brachial BP > 150/90 mmHg, consumed > 7 alcoholic drinks per week, and used tobacco, hormone replacement therapy, or dietary supplements with vasodilatory and/or antioxidant effects. The rationale for excluding women with a BP > 150/90 mmHg and > 7 alcoholic drinks was the increased CVD risk associated with these levels [35,36]. The study was registered in ClinicalTrials.gov (Identifier: NCT04672447) and the protocol was approved by the Texas Tech University Institutional Review Board.

### 2.2. Experimental Protocol

**Figure 1:**
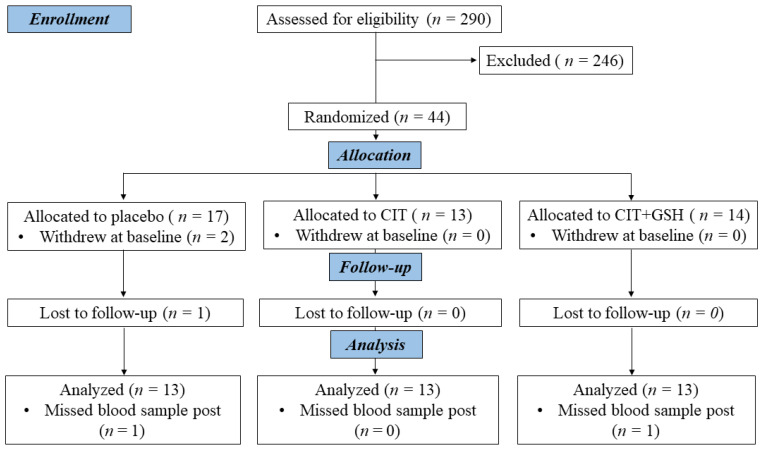
CONSORT flow chart of participants through the study. CIT, citrulline, CIT+GSH, and CIT+glutathione.

This was a double-blind, placebo-controlled, parallel design study. The randomization was performed by a researcher not involved in laboratory measurements using a block scheme stratified by age and systolic BP (SBP) with a computer program [37]. Forty-four participants were randomly assigned to a daily supplementation with a placebo (crystalline cellulose) (*n* = 17), CIT (6 g) (*n* = 13) or CIT+GSH (2 g + 200 mg: Setria^®^) (*n* = 14) (Figure 1). Participants consumed 9 capsules in the morning and evening for 4 weeks (Kyowa Hakko Bio Co., Ltd., Tokyo, Japan). Each capsule containing CIT, CIT+GSH, and placebo was indistinguishable by size, shape, and taste. Both research staff and participants were blinded to the group allocation until the completion of data analysis. Participants were instructed to avoid ARG or CIT rich containing foods (e.g., watermelon, salmon, nuts [walnuts, almonds], turkey breast) or supplements with NO precursors or antioxidants during the study period. Participants were recommended to maintain their usual diet and physical activity throughout the study. The last capsules were consumed 10–12 h before their laboratory visit at the end of the study. Participants returned their bottles during the last visit and adherence was assessed by capsule count.

The primary endpoint was the change in endothelial function, as assessed by brachial FMD. The secondary endpoints were reductions in PWV at rest and BP responses to CPT. Tertiary endpoints were changes in serum glucose, insulin, ARG, CIT, ORN, ADMA, arginase I, and oxidative markers.

Participants completed a health-history questionnaire and were familiarized with the protocol. Height and weight were measured using a stadiometer and beam scale (free-standing portable height rod and weigh beam, Detecto, Webb City, MO, USA), and waist circumference was measured using a non-elastic tape. Body mass index was calculated as weight (kg) divided by height squared (m^2^) and body composition was assessed using a total body dual-energy X-ray absorptiometry scanner (GE Lunar Prodigy; GE Healthcare, Madison, WI, USA).

### 2.3. Measurements

All measurements were performed in the morning after an overnight fast and 24 h abstinence from caffeine, alcohol, prescription medications, and physical activity. All vascular measurements were conducted in the supine position after at least 20 min of rest in a quiet, dimly lit, and thermoneutral environment. Following 4 weeks of intervention, all measurements were repeated in the same order and conducted at baseline.

#### 2.3.1. Flow-Mediated Dilation

Endothelial function was assessed by brachial FMD. The right brachial artery was scanned ~2–3 cm proximal to the antecubital fossa using a 12-MHz linear array Doppler ultrasound probe (LogiQ S7 expert, GE Medical Systems, Milwaukee, WI, USA) at an insonation angle < 60°. Following a 2 min baseline diameter and mean blood velocity recording, the occlusion cuff on the proximal forearm was rapidly inflated to 250 mmHg using an automated pump (E20, Hokanson, Bellevue, WA, USA). Following a 5 min occlusion period, the cuff was rapidly deflated while the diameter and mean blood velocity were continuously recorded for 3 min using open-source software (OBS Studio). Baseline and peak diameters were measured using an automated edge-detection software (Quipu Cardiovascular Suite, Pisa, Italy). Baseline diameter was measured as the average vessel diameter during the 2 min baseline, and peak diameter as the largest diameter detected post-occlusion. FMD is expressed as a percentage change from the baseline diameter: FMD% = ([peak diameter − baseline diameter]/baseline diameter) × 100.

#### 2.3.2. Pulse Wave Velocity

Carotid-femoral PWV (cfPWV, aortic), carotid-radial PWV (crPWV, brachial), carotid-distal PWV (cdPWV, systemic), and femoral-ankle (dorsalis pedis artery) PWV (faPWV, leg) were determined by applanation tonometry (Complior Analyse, Alam Medical, Vincennes, France; for faPWV: SphygmoCor CPV, AtCor Medical, Sydney, Australia). Distances between arterial sites in each segment were measured with a segmometer (Mitutoyo, Aurora, IL, USA). PWV was calculated as the distance of the arterial segment divided by the transit time of the pulse. cfPWV was multiplied by 0.80 to account for the distance between the carotid artery site and the suprasternal notch [38]. A minimum of two PWV measurements were obtained and the average was used for analysis.

#### 2.3.3. Brachial and Aortic Blood Pressures at Rest and During the CPT

Brachial SBP and diastolic BP (DBP) were measured using an automated oscillometric device (HEM-705CP; Omron Healthcare, Vernon Hill, IL, USA). Radial artery pressure waves recorded using applanation tonometry were calibrated with brachial mean arterial pressure (MAP) and DBP. Aortic hemodynamics were obtained via a validated transfer function (SphygmoCor CPV; AtCor Medical, Sydney, Australia). A minimum of two high-quality readings (operator index ≥ 80%) were obtained at rest and during the CPT and the average was used for analysis. The participants introduced their right hand up to the wrist in cold water at 1–4 °C for 2 min. The increase in BP from rest to the second minute of the CPT (Δ) was used for the analysis, since greater sympathetic activity occurs at this time point [16].

#### 2.3.4. Serum Biomarkers

Fasted blood samples were collected from the antecubital vein at baseline and at 4 weeks. Serum samples were collected using silicone-coated tubes and stored at −80 °C until subsequent analysis of the biomarkers. Glucose (EIAGLUC; Invitrogen, Carlsbad, CA, USA) was measured using the glucose oxidase method. Insulin was measured using an ELISA kit (80-INSHUE01.1, ALPCO Diagnostics, Salem, NH, USA). The homeostatic model assessment for insulin resistance was calculated as [fasting glucose (mg/dL) × fasting insulin (μIU/mL)]/405. Glutathione peroxidase (ab102530; Cambridge, UK), malondialdehyde (Abcam 233571; Cambridge, UK), and superoxide dismutase (ab119520; Cambridge, UK) were analyzed using the colorimetric method. Arginase-1 (BMS2216; Invitrogen, Carlsbad, CA, USA) and oxidized low-density lipoprotein (CSB-E07931h; Cusabio Biotech, Houston, TX, USA) were measured using ELISA kits. Serum ARG, CIT, ORN, and ADMA were analyzed using an AbsoluteIDQ p400 HR kit (Biocrates, Innsbruck, Austria), which is a combined flow injection and high-performance liquid chromatography–tandem mass spectrometry assay. The tandem mass spectrometry platform consisted of a Thermo Scientific Vanquish HPLC coupled to the Thermo Scientific Q-Exactive HF Quadrupole-Obitrap mass spectrometer.

### 2.4. Statistical Analysis

Sample size was estimated based on data that showed increased FMD after ARG supplementation in older adults [21]. It was estimated that 12 participants would be appropriate to detect a difference with ≥80% power at the α = 0.05 level. The Shapiro–Wilk test was used to verify the normal distribution of the data. A one-way analysis of variance was used to detect between-group differences. A two-way analysis of variance with repeated measures and Bonferroni adjustments were performed to detect time (before and after) and between-groups (placebo, CIT and CIT+GSH) differences in measures at rest and BP responses (Δ) to CPT. If significant time-by-group interactions were detected, Tukey and paired-t tests were used as post-hoc tests. Pearson’s correlation coefficient was used to evaluate relationships between changes in FMD% from 0 to 4 weeks and baseline FMD, and with aortic MAP responses to CPT from 0 to 4 weeks. Independent sample t-tests were used to evaluate low (0.20), moderate (0.50), and large (≥0.80) effect sizes using Cohen’s d on the changes in FMD between CIT vs. placebo, CIT+GSH vs. CIT, and CIT+GSH vs. placebo. Statistical analyses were performed using SPSS Ver26 (IBM SPSS, Armonk, NY, USA). Results are reported as the mean ± standard deviation (SD) in tables and the mean ± standard error (SE) in figures. Significance was set a priori at *p* < 0.05.

## 3. Results

### 3.1. Participant Characteristics

Participants were recruited from June 2020 to December 2021. Thirty-nine participants randomized to the placebo (*n* = 13), CIT (*n* = 13), and CIT+GSH (*n* = 13) completed the study (Figure 1). Compliance to the supplements were 94.9 ± 1.3%, 95.7 ± 1.1%, and 95.5 ± 1.2% for placebo, CIT, and CIT+GSH groups, respectively. No adverse effects of the supplementations were reported by participants during the study.

Participant characteristics at baseline did not differ among the groups, except for FBG (Table 1). FBG was higher in CIT+GSH compared to the placebo but not to CIT (*p* = 0.29).

### 3.2. Flow-Mediated Dilation and Arterial Stiffness

**Figure 2:**
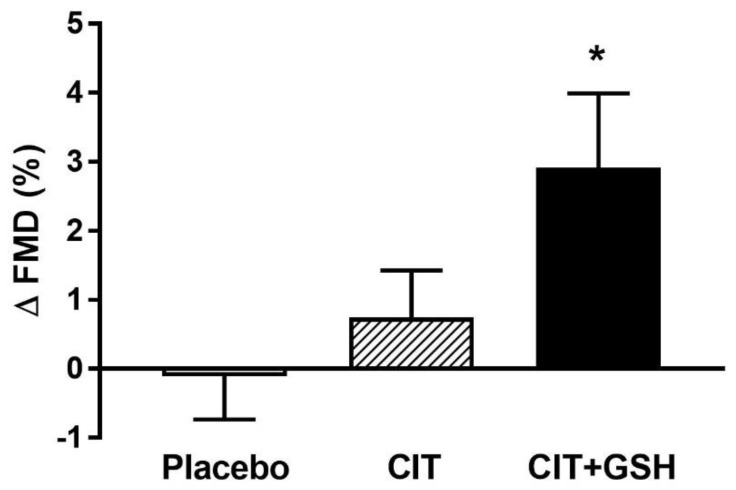
Changes (Δ) in brachial artery flow-mediated dilation (ΔFMD%) from 0 to 4 weeks. Abbreviations: CIT, citrulline; CIT+GSH, CIT+glutathione. * *p* < 0.05 vs. placebo.

**Figure 3:**
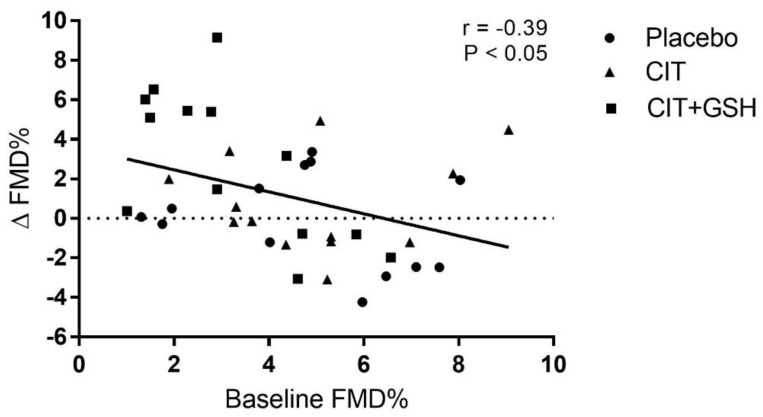
Relationship between changes (Δ) in the brachial artery flow-mediated dilation (ΔFMD%) from 0 to 4 weeks and baseline (0 week) FMD%. Abbreviations: CIT, citrulline; CIT+GSH, CIT+glutathione.

Baseline FMD and PWV segments did not differ among the groups. There was a time-by-group interaction for FMD. CIT+GSH increased the FMD by 2.9% (*p* = 0.045) compared with placebo, but not with CIT (*p* = 0.18) (Figure 2). A low effect size was seen between CIT and placebo (*d* = 0.32) and, although insignificant (*p* = 0.10), there was a moderate effect size between CIT+GSH and CIT (*d* = 0.67). To compliment the significant increase in FMD, there was a high effect size between CIT+GSH and placebo (*d* = 0.91, *p* = 0.03). The change in FMD from 0–4 weeks was correlated with the baseline FMD (Figure 3). The Pearson correlation coefficient for placebo, CIT, and CIT+GSH were −0.26 (*p* = 0.39), 0.16 (*p* = 0.60), and −0.68 (*p =* 0.01), respectively. There were no significant time-by-group interactions for cfPWV, crPWV, cdPWV, or faPWV. However, CIT+GSH supplementation decreased crPWV (arm PWV) by 0.66 ± 0.93 m/s (*p* = 0.05, Table 2).

### 3.3. Blood Pressure at Rest and During the Cold Pressor Test

**Figure 4:**
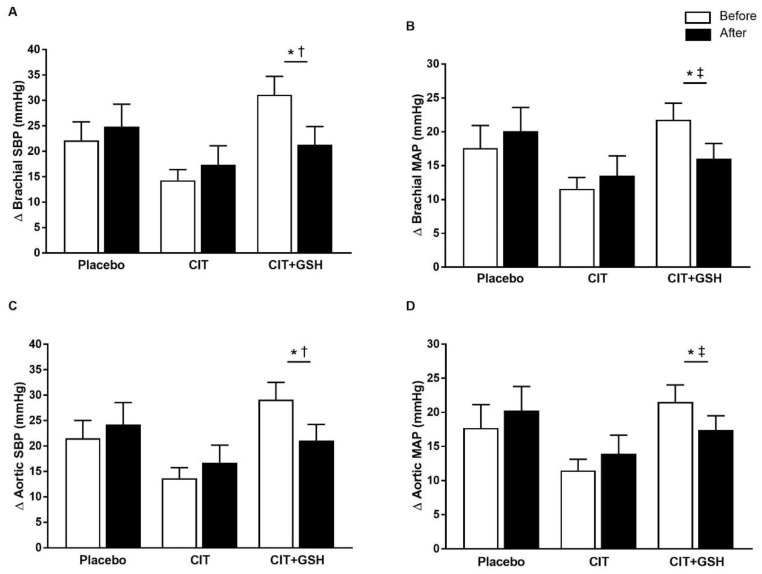
Changes (Δ) in (**A**) the brachial systolic blood pressure SBP (SBP), (**B**) brachial mean arterial pressure (MAP), (**C**) aortic SBP, and (**D**) aortic MAP to the cold pressor test before and after supplementations. Abbreviations: CIT, citrulline; CIT+GSH, CIT+glutathione. * *p* < 0.05 before vs. after, ^†^ *p* < 0.05 vs. placebo and CIT, ^‡^ *p* < 0.05 vs. placebo.

Table 3 shows the BP at rest and during the CPT before and after the supplementations. There were no significant between-group differences in the resting BP at baseline and no time-by-group interactions. There were significant time-by-group interactions for changes (Δ) in brachial SBP (*p* = 0.02), brachial MAP (*p* = 0.04), aortic SBP (*p* = 0.03), and aortic MAP (*p* = 0.04) responses to the CPT. CIT+GSH supplementation reduced ΔSBP compared to the placebo and CIT (*p* < 0.05 for both), and reduced ΔMAP compared to the placebo (*p* < 0.05) but not to CIT (Figure 4). No significant time-by-group interactions were observed for ΔDBP, Δ augmentation index normalized to a heart rate of 75, and Δ reflection time (Tr). Attenuation of aortic ΔMAP was significantly related to the improvement in FMD% (*r* = −0.33, *p* < 0.05).

### 3.4. Serum Biomarkers

**Figure 5:**
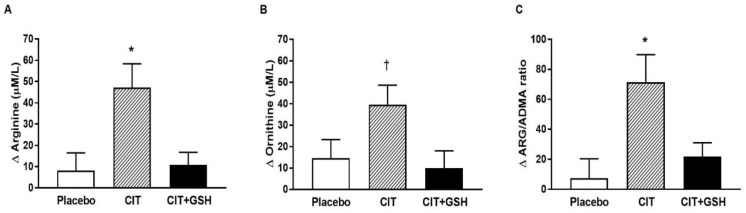
Changes (Δ) in serum levels of arginine (**A**), ornithine (**B**), and the arginine/ADMA ratio (**C**) from 0–4 weeks in the three groups. Values are the mean ± SE. Abbreviations: ARG, arginine; ADMA, asymmetric dimethylarginine; CIT, citrulline; CIT+GSH, CIT+glutathione. * *p* < 0.05 vs. placebo and CIT+GSH; ^†^ *p* < 0.05 vs. CIT+GSH.

Table 4 shows serum biomarkers before and after the interventions. Significant time-by-group interactions were observed for ARG (*p* = 0.005), ORN (*p* = 0.04), and ARG/ADMA ratio (*p* = 0.007). ARG and ARG/ADMA ratios were significantly increased by CIT supplementation compared with placebo (*p* = 0.01 for both) and CIT+GSH (*p* = 0.008 and *p* = 0.04, respectively) (Figure 5A,D). The ARG/ADMA ratio significantly increased after CIT+GSH (*p* = 0.04). ORN was increased after CIT compared to CIT+GSH (*p* = 0.05) but not to the placebo (*p* = 0.12). Arginase I levels decreased after CIT (*p* = 0.01) and tended (*p* = 0.07) to decrease after CIT+GSH, but there was no significant time-by-group interaction. Glucose, insulin, homeostatic model assessment for insulin resistance, glutathione peroxidase, superoxide dismutase, oxidized LDL, and malondialdehyde did not significantly change after 4 weeks in any group.

## 4. Discussion

This study examined the effects of 4 weeks of CIT and CIT+GSH supplementation on vascular function and BP responsiveness in healthy postmenopausal women. We found that CIT+GSH supplementation improved endothelial function through an increase in the ARG/ADMA ratio. Although CIT supplementation increased serum ARG levels and the ARG/ADMA ratio, it did not statistically improve FMD. In addition, CIT+GSH supplementation attenuated BP responsiveness to CPT. These data show that CIT+GSH supplementation has protective cardiovascular effects at rest and during sympathetic stimulation (CPT) in healthy postmenopausal women.

FMD decreases progressively throughout the stages of the menopausal transition in healthy women [4]. Low FMD is a valuable biomarker for predicting future cardiovascular events in apparently healthy adults [39]. In this study, we showed that 4 weeks of CIT+GSH supplementation increased brachial FMD by 2.9% in healthy postmenopausal women. Consistent with our findings, a non-placebo controlled study observed a beneficial effect of CIT supplementation for 4 weeks on FMD in middle-aged patients with vasospastic angina and low FMD [40]. Similarly, 2 weeks of ARG supplementation increased FMD by 3.1% in healthy older men with age-related endothelial dysfunction [21]. A previous meta-analysis suggested that ARG supplementation improves FMD in individuals with baseline FMD values < 7.0% [41]. These previous findings suggest that CIT and ARG improve FMD in individuals with low FMD. Our data suggest that improvements in FMD following CIT+GSH mainly occurred in women with a low baseline FMD. Importantly, we found that the increase in FMD after 4 weeks of CIT+GSH was 0.91 SD greater vs. placebo and 0.67 SD greater vs. CIT alone. Given that for each 1 SD increase in FMD there is an associated 50% lower risk of cardiovascular events [42], our findings are clinically important.

Recent data showed that FMD values lower than 5.4% are indicative of impaired endothelial function in apparently healthy adults [43]. Based on this FMD reference value, 85% of women in the CIT+GSH group had low FMD, and thus, had impaired endothelial function at baseline. Evidence indicates that cardiometabolic risk factors, including BP and FBG, are determinants of low FMD [43]. Independent of traditional cardiovascular risk factors, low FMD predicts CVD risk [44]. In our study, an elevated baseline FBG was observed in the CIT+GSH group. Based on FBG values in the CIT+GSH group, four and seven participants had prediabetes or an increased risk of incident prediabetes, respectively [45]. It is known that prediabetes can negatively impact the age-related decline in FMD [46,47]. We demonstrate that CIT+GSH supplementation increased the mean FMD above 5.4% in a group of postmenopausal women with elevated FBG. Considering that a 4.3% increase in FMD is associated with 50% CVD risk reduction [42], the 2.8% improvement in FMD following 4 weeks of CIT+GSH may reduce the risk of cardiovascular events in apparently healthy postmenopausal women.

Age associated arterial stiffening affects the aorta to a greater extent than peripheral muscular arteries [48]. In older women, 10 years of aging have a greater impact on aortic PWV (+2.4 m/s) than brachial PWV (+0.19 m/s), indicating that peripheral arteries have less stiffening than the aorta [48]. A widely used measure of arterial stiffness is brachial-ankle PWV (baPWV), a segment that includes central (aortic PWV) and peripheral (brachial and leg PWV) arteries [49]. Since peripheral arteries have relatively more smooth muscle cells than collagen fibers [48], brachial PWV and leg PWV are more responsive to NO-mediated vasodilation than the aorta [50,51]. Oral CIT supplementation (5.6 g daily) for 1 week significantly reduced baPWV by ~0.14 m/s in middle-aged men with high baseline baPWV [27]. A further study clarified that the beneficial effect of CIT (6 g daily) on baPWV was due to a reduction of leg PWV (~0.40 m/s) with no effect on aortic PWV in obese postmenopausal women [29]. In the present study, aortic PWV was not reduced by CIT and CIT+GSH supplementations. This ineffectiveness may be attributed to the normal baseline values of our participants, which are considered to be ~7.6 m/s for adults aged ≥50 years [38]. Importantly, CIT+GSH decreased brachial PWV by ~0.40 m/s in postmenopausal women with increased risk of prediabetes. This finding is in agreement with previous studies [27,29] and confirms that peripheral arteries are more responsive than the aorta to dietary supplementation with NO precursors. The decrease in brachial PWV observed in the current study may be attributed to enhanced endothelial-mediated vasodilation [51].

Impaired FMD and increased sympathetic activity are associated with the increased risk of incident hypertension in healthy postmenopausal women [13,14]. Our participants had a normal or elevated SBP at rest. Our observation that the resting BP was unaffected by CIT and CIT+GSH supplementations is consistent with previous studies in normotensive adults [30,32] and middle-aged adults with elevated SBP and FBG [27]. Thus, we used the CPT as a systemic sympathetic vasoconstrictor stimulus to evaluate the efficacy of dietary supplements for attenuating BP reactivity to CPT [18,19]. We observed reductions of brachial and aortic SBP and MAP responses to CPT after CIT+GSH but not CIT supplementation. Previous evidence of blunted BP responsiveness to CPT after 2 weeks of CIT (6 g/day) was observed in healthy young [31] and older adults [32]. However, we did not measure FMD in those previous studies. In mice, CIT supplementation attenuated cold hypersensitivity by improving endothelial function [52]. In the current study, the attenuation of aortic MAP reactivity to CPT was related to improvements in FMD. This finding suggests that improved FMD with CIT+GSH supplementation may reduce the risk of cardiovascular events related to the augmented aortic BP load during conditions with increased sympathetic stimulation [16,19,53].

ADMA competes with ARG for binding to eNOS, thereby a decrease in ARG/ADMA leads to reduced NO production [54]. Evidence supports that the ARG/ADMA ratio is a better biomarker of endothelial function than circulating ARG and ADMA levels alone [7,55]. Our participants had normal ARG/ADMA values at baseline [56]. However, the CIT+GSH group tended to have a lower ARG/ADMA ratio, which has been associated with hyperglycemia [57]. In the present study, CIT and CIT+GSH supplementations increased the ARG/ADMA ratio, suggesting increased ARG availability for NO production [54]. This finding is consistent with Ochiai et al. [27] who reported an increase in plasma ARG/ADMA due to the isolated increase in ARG levels. CIT supplementation increases circulating ARG via de novo synthesis in the kidneys [54]. Serum ARG introduction to endothelial cells via the cationic amino acid y+ transporter 1 depends on both ARG and ADMA levels, since they compete for the transporter [58]. Therefore, a greater ARG availability displaces ADMA from eNOS binding, thereby improving NO synthesis [7,27,40,54,59]. A recent study reported that 2 g of CIT for 4 weeks increased NOx levels in type 2 diabetes patients as a result of arginase inhibition [23]. These findings suggest that a low dose of CIT may improve endothelial function in individuals with hyperglycemia. In the study by Ochiai et al., the increase in ARG/ADMA was evident after CIT supplementation in men with FBG in the prediabetes category [27]. Therefore, augmented ARG/ADMA ratio may explain the improvement in endothelial function following CIT and ARG supplementations in adults with hyperglycemia [27] and low baseline FMD [21,40,60].

Despite greater increases in the serum ARG levels and ARG/ADMA ratio, FMD did not improve after CIT supplementation alone. This discrepancy in the findings may be explained by the healthy status of the participants. In agreement with our findings, ARG supplementation for 4 weeks increased serum ARG levels by almost double but failed to improve FMD in healthy postmenopausal women [61]. Similarly, 6 g of CIT supplementation efficiently increased circulating ARG levels and ARG/ADMA without affecting FMD in healthy adults [24]. In the present study, the increase in ARG levels positively increased the ARG/ADMA ratio, indicating improved ARG availability for NO production. Similarly, previous studies failed to show improvement in endothelial function assessed as increased NOx after oral ARG and CIT supplementations in healthy adults [25,62]. Thus, increased ARG availability via CIT or ARG supplementation may not improve endothelial function in individuals with normal FMD [24,61]. Of note, CIT is concurrently produced with NO by eNOS, and recycled to de novo ARG [63]. It is possible that CIT supplementation stimulated the production of NO and CIT, but the effect on FMD was not evident in the absence of endothelial dysfunction. Nonetheless, the increase in the ARG/ADMA ratio by CIT may have vascular benefits, since an increase in this ratio by 1 SD may decrease CVD risk by 20% [64].

Oxidative stress markers were not improved by both supplementations. In healthy adults, 200–1000 mg of GSH daily for 4 weeks did not improve microvascular endothelial function and malondialdehyde levels [65,66]. Recently, whole blood GSH was elevated after 1 month with 250 or 1000 mg of GSH supplementation [67]. Despite no improvement in the oxidative stress markers, CIT+GSH increased FMD by improving eNOS function via increased ARG availability [33]. The ability of CIT+GSH to increase NO production was demonstrated in human umbilical vein endothelial cells after exposure for 24 h to the combination but not to CIT and GSH alone [34]. Thus, GSH provides an augmenting effect to the conversion of ARG to NO [34].

There are some limitations in the present study. The sample size was relatively small and included healthy postmenopausal women. FBG was not considered for randomization, which resulted to be higher in the CIT+GSH group. Future studies might investigate the effects of CIT and CIT+GSH in individuals with prediabetes. A GSH dose greater than 200 mg daily for longer than 4 weeks of supplementation may reduce markers of oxidative stress, as previously shown [67]. We measured serum levels of superoxide dismutase and glutathione peroxidase, two intracellular enzymes, rather than intracellular enzymatic activity. A statistical limitation could be a “regression to the mean” phenomenon when examining changes in FMD from baseline. However, there were no significant decreases in FMD in the placebo group after the 4-week intervention, which supports our conclusion of CIT+GSH being a viable route to improve endothelial function in healthy postmenopausal women.

## 5. Conclusions

Four weeks of CIT+GSH supplementation improves brachial artery FMD in apparently healthy postmenopausal women with slightly elevated FBG. CIT+GSH reduced brachial PWV and attenuates BP responses to sympathetic activation. These beneficial effects on the endothelial function and BP reactivity may be attributed to an increased ARG/ADMA ratio. Therefore, CIT+GSH supplementation may reduce the risk of cardiovascular events during physiological stress in postmenopausal women. Although CIT supplementation alone caused greater increases in the serum ARG levels and ARG/ADMA ratio, it did not improve FMD or BP responses to CPT in healthy postmenopausal women.

## Figures and Tables

**Table 1:** Participant characteristics and treatments.

Measures and Medication	Placebo (*n* = 13)	CIT (*n* = 13)	CIT+GSH (*n* = 13)	*p*
Age (years)	60 ± 5	58 ± 4	58 ± 6	0.60
Height (m)	1.58 ± 0.06	1.59 ± 0.08	1.59 ± 0.10	0.92
Body weight (kg)	73.3 ± 9.3	72.1 ± 11.2	75.5 ± 15.7	0.94
BMI (kg/m^2^)	29.3 ± 3.4	29.0 ± 4.8	29.5 ± 4.2	0.91
WC (cm)	93.3 ± 8.5	93.1 ± 12.6	91.5 ± 11.0	0.65
Total fat mass (%)	45 ± 5	42 ± 7	46 ± 5	0.18
Total lean mass (%)	54 ± 5	55 ± 5	56 ± 6	0.18
FBG (mg/dL)	87 ± 7	90 ± 6	94 ± 7 ^†^	0.04
**Medication (*n*)**				
ARB/ACE inhibitors	2	1	3	
Diuretics	1	0	2	
Ca^2+^ channel blockers	1	0	0	

Values are the mean ± SD or number (*n*) of participants. Abbreviations: BMI, body mass index; WC, waist circumference; FBG, fasting blood glucose; CIT, citrulline; GSH, glutathione; ARB, angiotensin receptor blocker; ACE, angiotensin converting enzyme; ^†^ vs. placebo.

**Table 2:** Brachial artery endothelial function and arterial stiffness.

Measure	Placebo		CIT		CIT+GSH		
	Before	After	Before	After	Before	After	*p*
FMD (%)	4.8 ± 2.2	4.8 ± 2.9	5.0 ± 2.0	5.7 ± 3.5	3.1 ± 1.8	6.0 ± 3.0 *^‡^	0.04
cfPWV (m/s)	7.6 ± 1.4	7.6 ± 1.1	6.9 ± 0.8	7.3 ± 0.8	7.3 ± 0.7	7.3 ± 1.3	0.28
crPWV (m/s)	8.3 ± 1.7	8.3 ± 1.2	8.5 ± 1.2	8.5 ± 1.8	8.5 ± 0.9	7.9 ± 0.9 *	0.51
cdPWV (m/s)	8.9 ± 1.5	8.2 ± 1.1	8.8 ± 0.8	8.4 ± 0.9	8.2 ± 0.9	8.2 ± 1.1	0.23
faPWV (m/s)	8.5 ± 0.3	8.7 ± 0.3	9.0 ± 0.3	9.3 ± 0.3	9.0 ± 0.3	8.8 ± 0.3	0.69

Values are the mean ± SD. Abbreviations: cfPWV, carotid-femoral PWV; crPWV, carotid-radial PWV; cdPWV, carotid-distal PWV; CIT, citrulline; CIT+GSH, CIT+glutathione; faPWV, femoral-ankle PWV. *p*-values are the time-by-group interaction from two-way repeated measures ANOVA. * *p* < 0.05 vs. before; ^‡^ *p* < 0.05 vs. placebo.

**Table 3:** Brachial and aortic blood pressures at rest and during CPT.

Measure	Condition	Placebo		CIT		CIT+GSH		
		Before	After	Before	After	Before	After	*p*
Brachial SBP (mmHg)	Rest	113 ± 13	111 ± 12	113 ± 13	114 ± 11	110 ± 12	115 ± 12	0.27
	CPT	135 ± 15	136 ± 12	127 ± 13	132 ± 14	141 ± 18	135 ± 16	
Brachial DBP (mmHg)	Rest	75 ± 8	75 ± 8	71 ± 9	71 ± 9	71 ± 11	73 ± 8	0.69
	CPT	91 ± 11	93 ± 8	81 ± 9	83 ± 8	88 ± 14	89 ± 10	
Brachial MAP (mmHg)	Rest	88 ± 9	87 ± 9	85 ± 10	85 ± 7	84 ± 11	87 ± 7	0.40
	CPT	105 ± 11	107 ± 9	96 ± 9	99 ± 9	106 ± 14	103 ± 11	
Aortic SBP (mmHg)	Rest	107 ± 13	105 ± 11	108 ± 13	109 ± 10	104 ± 11	107 ± 9	0.35
	CPT	129 ± 15	129 ± 12	121 ± 14	125 ± 13	133 ± 18	128 ± 16	
Aortic DBP (mmHg)	Rest	76 ± 8	76 ± 8	72 ± 9	72 ± 9	72 ± 11	74 ± 8	0.66
	CPT	92 ± 11	94 ± 8	82 ± 9	83 ± 8	90 ± 14	90 ± 10	
Aortic MAP (mmHg)	Rest	87 ± 9	86 ± 9	84 ± 10	84 ± 8	83 ± 11	85 ± 7	0.70
	CPT	104 ± 11	106 ± 9	95 ± 10	97 ± 9	104 ± 14	102 ± 11	
AIx75 (%)	Rest	27 ± 6	28 ± 7	30 ± 8	29 ± 8	27 ± 5	27 ± 6	0.88
	CPT	33 ± 6	31 ± 7	32 ± 8	32 ± 6	33 ± 8	30 ± 7	
Tr (ms)	Rest	137 ± 6	138 ± 7	135 ± 8	135 ± 8	138 ± 9	136 ± 7	0.29
	CPT	135 ± 8	139 ± 11	135 ± 8	136 ± 10	138 ± 8	139 ± 8	

Values are the mean ± SD. Abbreviations: AIx, augmentation index normalized to the heart rate of 75; CIT, citrulline; CIT+GSH, CIT+glutathione; DBP, diastolic blood pressure; MAP, mean arterial pressure; SBP, systolic blood pressure; Tr, reflection time. *p*-values of ANOVA time-by-group interaction.

**Table 4:** Serum markers of arginine metabolism, glucose control, and oxidative stress.

Measure	Placebo		CIT		CIT+GSH		
	Before	After	Before	After	Before	After	*p*
Arginine (µM/L)	127 ± 21	135 ± 32	121 ± 21	168 ± 36 *^†^	117 ± 25	128 ± 19	0.005
Citrulline (µM/L)	41 ± 10	41 ± 13	40 ± 8	49 ± 13 *	38 ± 10	39 ± 7	0.09
Ornithine (µM/L)	106 ± 28	120 ± 34	110 ± 20	149 ± 38 ^‡#^	104 ± 25	114 ± 24	0.043
ADMA (mM/L)	0.54 ± 0.05	0.55 ± 0.05	0.54 ± 0.06	0.56 ± 0.06 *	0.55 ± 0.07	0.54 ± 0.08	0.27
Arginine/ADMA	240 ± 50	248 ± 61	228 ± 50	299 ± 48 *^†^	215 ± 47	237 ± 37 *	0.007
Arginase-I (ng/mL)	87 ± 2	87 ± 2	88 ± 3	87 ± 3 ^‡^	90 ± 3	89 ± 3	0.32
Glucose (mg/dL)	87 ± 7	87 ± 7	90 ± 6	91 ± 10	94 ± 7	94 ± 8	0.89
Insulin (mIU/mL)	15 ± 4	14 ± 4	15 ± 6	15 ± 5	12 ± 4	12 ± 5	0.75
HOMA-IR	3.1 ± 1.0	3.1 ± 1.1	3.4 ± 1.5	3.4 ± 1.3	2.7 ± 1.1	2.9 ± 1.1	0.82
GPx (µmol/L)	393 ± 44	399 ± 44	396 ± 43	400 ± 43	435 ± 35	439 ± 33 *	0.75
SOD (ng/mL)	20 ± 3	20 ± 2	21 ± 3	20 ± 2	21 ± 4	20 ± 3	0.42
Ox-LDL (U/L)	65 ± 13	65 ± 14	69 ± 12	68 ± 12	72 ± 12	72 ± 12	0.53
MDA (µm/L)	1.0 ± 0.37	1.1 ± 0.39	0.94 ± 0.39	0.99 ± 0.38	1.1 ± 0.45	1.0 ± 0.38	0.76

Data are the mean ± SD. *p*-values of ANOVA time-by-group interaction. Abbreviations: ADMA, asymmetric dimethylarginine; HOMA-IR, homeostatic model assessment for insulin resistance; GPx, glutathione peroxidase; SOD, superoxide dismutase; Ox-LDL, oxidized LDL; MDA, malondialdehyde. * *p* < 0.05 vs. before; ^‡^ *p* < 0.01 vs. before; ^†^ *p* < 0.05 vs. placebo and CIT+GSH; ^#^ *p* < 0.05 vs. CIT+GSH.

## Data Availability

The data presented in this study are available upon request from the corresponding author.

## Abbreviations

ADMA	Asymmetric dimethylarginine
ARG	L-Arginine
baPWV	Brachial-ankle pulse wave velocity
BP	Blood pressure
cdPWV	Carotid-distal pulse wave velocity
cfPWV	Carotid-femoral pulse wave velocity
CIT	L-citrulline
CPT	Cold pressor test
crPWV	Carotid-radial pulse wave velocity
CVD	Cardiovascular disease
DBP	Diastolic blood pressure
eNOS	Endothelial nitric oxide synthase
faPWV	Femoral-ankle pulse wave velocity
FBG	Fasting blood glucose
FMD	Flow-mediated dilation
GSH	Glutathione
MAP	Mean arterial pressure
NO	Nitric oxide
NOx	NO metabolites
ORN	Ornithine
PWV	Pulse wave velocity
SBP	Systolic blood pressure
SD	Standard deviation
SE	Standard error
